# Risk factors for esophageal stricture after circumferential endoscopic submucosal dissection: a preliminary, multicenter, retrospective, case-control study

**DOI:** 10.3389/fmed.2026.1826298

**Published:** 2026-06-11

**Authors:** Yang Bai, Wei-Xing Yang, Min Qiao, Xin Yang

**Affiliations:** 1 Department of Respiratory and Critical Care Medicine, The First Affiliated Hospital of Chongqing Medical University, Chongqing, China; 2 Department of Gastroenterology, The Affiliated Hospital of Southwest Medical University, Luzhou, China; 3 Department of Clinical Nutrition, The First Affiliated Hospital of Chongqing Medical University, Chongqing, China; 4 Department of Gastroenterology, The First Affiliated Hospital of Chongqing Medical University, Chongqing, China

**Keywords:** endoscopic submucosal dissection, esophageal stricture, prophylactic measure, risk factor, superficial esophageal cancer

## Abstract

**Background:**

Post-circumferential endoscopic submucosal dissection (cESD) esophageal stricture remains a refractory complication despite combined steroid therapy (CST). The submucosal steroid pre-injection strategy (SSPS) has emerged as a novel prophylactic approach. This study aimed to preliminarily identify risk factors for post-cESD stricture, with a focus on steroid-based prophylaxis.

**Methods:**

This multicenter, retrospective, case-control study utilized the same patient dataset as our previously published cohort study and included 55 patients who underwent cESD with either SSPS or CST prophylaxis across eight tertiary hospitals in China (January 2022–August 2024). Univariate and multivariate logistic regression analyses were performed to identify risk factors by calculating odds ratios (ORs) with 95% confidence intervals (CIs), with subgroup analyses stratified by prophylactic measures.

**Results:**

The stricture and non-stricture groups comprised 28 and 27 patients, respectively. Baseline demographics, lesion features, longitudinal length, and transverse width of resected specimens were comparable between stricture and non-stricture groups (all *P* > 0.05). Multivariate analysis revealed that a longitudinal length of circumferential involvement exceeding 50 mm was an independent risk factor (OR 9.10, 95% CI 2.27–33.30, *P* = 0.002), while SSPS demonstrated a significant protective effect compared with CST (OR 0.22, 95% CI 0.05–0.91, *P* = 0.036). Subgroup analysis showed that extensive circumferential involvement remained associated with stricture in the CST group (*P* = 0.012) but not in the SSPS group (*P* = 0.268). Of note, the subgroup analysis was limited by small sample size, and these findings should be considered preliminary. Total steroid exposure was not calculated but substantially lower in SSPS than in CST, supporting a favorable risk–benefit profile for SSPS.

**Conclusion:**

A Longitudinal length of circumferential involvement exceeding 50 mm independently predicts post-cESD stricture, whereas SSPS provides superior protection compared to CST. These findings are preliminary and require validation in large-scale prospective studies. SSPS may potentially mitigate stricture risk in high-risk lesions, but its role in expanding cESD indications for extensive superficial esophageal cancer needs further exploration.

## Introduction

Esophageal cancer ranks as the sixth leading cause of cancer-related mortality globally, with prompt diagnosis and intervention markedly improving prognosis (1). Endoscopic submucosal dissection (ESD) has become a standard minimally invasive procedure for superficial esophageal cancer, with significant curative potential (2, 3). Esophageal stricture after extensive ESD, especially circumferential ESD (cESD), continues to be a refractory challenge (4). Notwithstanding the use of combined steroid therapy (CST), which comprises single intralesional triamcinolone acetonide (TA) injections followed by an 8-weeks tapering course of oral prednisolone, the incidence of post-cESD esophageal stricture remains at a high level, even potentially approaching 100% (5–8). This condition often requires multiple sessions of endoscopic balloon dilation, which impairs quality of life and risks perforation (9). We have introduced the submucosal steroid pre-injection strategy (SSPS), an innovative method that incorporates TA administration into the cESD procedure. This method entails the TA pre-injection to form a submucosal cushion before lesion dissection, followed by two postoperative intralesional TA injections on post-cESD days 5 and 12 to enhance anti-inflammatory efficacy (10). Our previous multicenter cohort study confirmed that SSPS significantly reduced the risk of post-cESD stricture compared to CST by providing an earlier, more uniform, and prolonged anti-inflammatory efficacy (11).

Several studies have sought to identify risk factors for post-ESD esophageal stricture. The extent of circumferential involvement has been recognized as an independent risk factor (12, 13). The steroids have been widely investigated as a potential prophylactic measure, but their efficacy remains uncertain, especially in those receiving cESD (5–8). Accurate identification of these risk factors is crucial to strengthen patient selection, guide clinical practice, formulate targeted prophylactic strategies, and optimize postoperative outcomes. This multicenter, retrospective, case-control study, using the same dataset (11), seeks to thoroughly investigate the risk factors associated with post-cESD esophageal stricture, emphasizing the impact of steroid-based prophylactic measures, thereby providing preliminary evidence for improving patient management in cESD contexts. We hypothesized that (1) longitudinal length of circumferential involvement over 50 mm is an independent risk factor for post-cESD stricture based on previous clinical studies and guidelines (14, 15), and (2) SSPS has a stronger protective effect against stricture formation compared to CST, particularly in high-risk lesions (10, 11).

## Materials and methods

### Study design and patients

This multicenter, retrospective, case-control study encompassed patients with or without post-cESD esophageal stricture from eight tertiary hospitals in China (from January 2022 to August 2024), using the same dataset from the previous multicenter cohort study (11). The study (adhering to the World Medical Association’s Declaration of Helsinki) was approved by the Ethics Committee of the First Affiliated Hospital of Chongqing Medical University (ZZ2024-226-01), and informed consent was waived due to its retrospective design.

Patients who underwent cESD for superficial esophageal cancer and received either SSPS or CST as prophylaxis against post-cESD stricture were included in this study. This study concentrated on cESD and steroid-based prophylactic strategies, excluding cases without SSPS or CST prophylaxis and other risk factors for esophageal stricture, such as chemoradiotherapy, additional esophageal surgery unrelated to stricture, local suturing of the post-cESD mucosal defect, and prior radiotherapy. The stricture group (case group) consisted of patients with esophageal stricture (inability to pass a 9.9 mm endoscope) confirmed by endoscopy 3 months post-cESD (16). The non-stricture group (control group) consisted of patients who underwent cESD during the same period and did not develop an esophageal stricture 3 months post-cESD. An unmatched design was applied due to the relatively small sample size of enrolled patients and the high incidence of post-cESD esophageal stricture (about 50%), complicating the matching process.

### Steroid prophylaxis protocols

SSPS: TA (40 mg/mL every 3 cm of the longitudinal length of the circumferential mucosal defect) pre-injection into the submucosa before dissection. Two additional local TA injections (2 mg per puncture, 1 cm apart) into the submucosal layer within the mucosal defect were administered on post-cESD days 5 and 12. No oral steroids were given (10).

CST: Single local TA injection (5 mg per puncture, 1 cm apart) immediately after dissection into the submucosal layer within the mucosal defect, and followed by an 8-weeks tapering oral prednisolone regimen: 30, 30, 25, 25, 20, 15, 10, and 5 mg for 7 days each (8, 11).

Total steroid exposure: SSPS: total local TA (no systemic steroids). CST: local TA + cumulative oral prednisolone 1120 mg.

### Data collection

Data were collected retrospectively from electronic medical records using standardized data abstraction forms. The choice of risk and protective factors to investigate was based on an extensive review of the literature (12, 13). Potential confounders considered included age, sex, alcohol consumption, cigarette consumption, family history of esophageal cancer, lesion location, macroscopic type, specimen size (longitudinal length and transverse width), pathological invasion depth, and prophylactic measure type (SSPS vs. CST). Macroscopic type was classified based on endoscopic appearance, primarily guided by the Japanese Classification of Esophageal Cancer (17). The whole specimen from en bloc resection was longitudinally sliced, flattened, and assessed for the aforementioned lesion features. The longitudinal length and transverse width of the resected specimen were evaluated at the largest longitudinal and transverse dimensions, respectively. The longitudinal length of circumferential involvement was determined by measuring the portion of the longitudinally sliced specimen that encompassed the entire esophageal circumference. The Japanese Classification of Esophageal Cancer was adopted to classify the pathological invasion depth (17). Dual independent data extraction was performed, followed by cross-verification. Discrepancies were reconciled by examining the original medical records, endoscopic reports, and pathology sections and discussed by two independent experts (Min Qiao and Xin Yang). The final data consistency rate was ≥95%, and the error rate for discrepancies was <5%.

### Statistical analyses

Statistical analyses were performed with R software version 4.3.0 (R Foundation). Categorical variables were summarized as frequencies, whilst continuous variables were expressed as means ± standard deviations for normally distributed data or as medians with interquartile ranges for skewed data. Group comparisons were conducted using the Pearson χ^2^ test or Fisher’s exact test for categorical data, the independent *t*-test for normally distributed continuous variables, and the Mann-Whitney U test for non-normally distributed variables.

Odds ratios (ORs) and 95% confidence intervals (CIs) were obtained using unconditional logistic regression models. A forced inclusion strategy based on clinical significance was adopted for multi-factor logistic regression. A subgroup analysis was conducted to evaluate the relationship between prophylactic measures (SSPS or CST) and lesion size, such as longitudinal length over 50 mm, transverse width over the average (established as the cutoff value), and longitudinal length of circumferential involvement over 50 mm. The cutoff value (50 mm) for the longitudinal length of circumferential involvement was based on previous clinical studies and guidelines (14, 15). All essential variables for enrolled patients were complete, necessitating no processing of missing data. A two-sided *P*-value < 0.05 was considered statistically significant.

## Results

### Baseline characteristics

**FIGURE 1:**
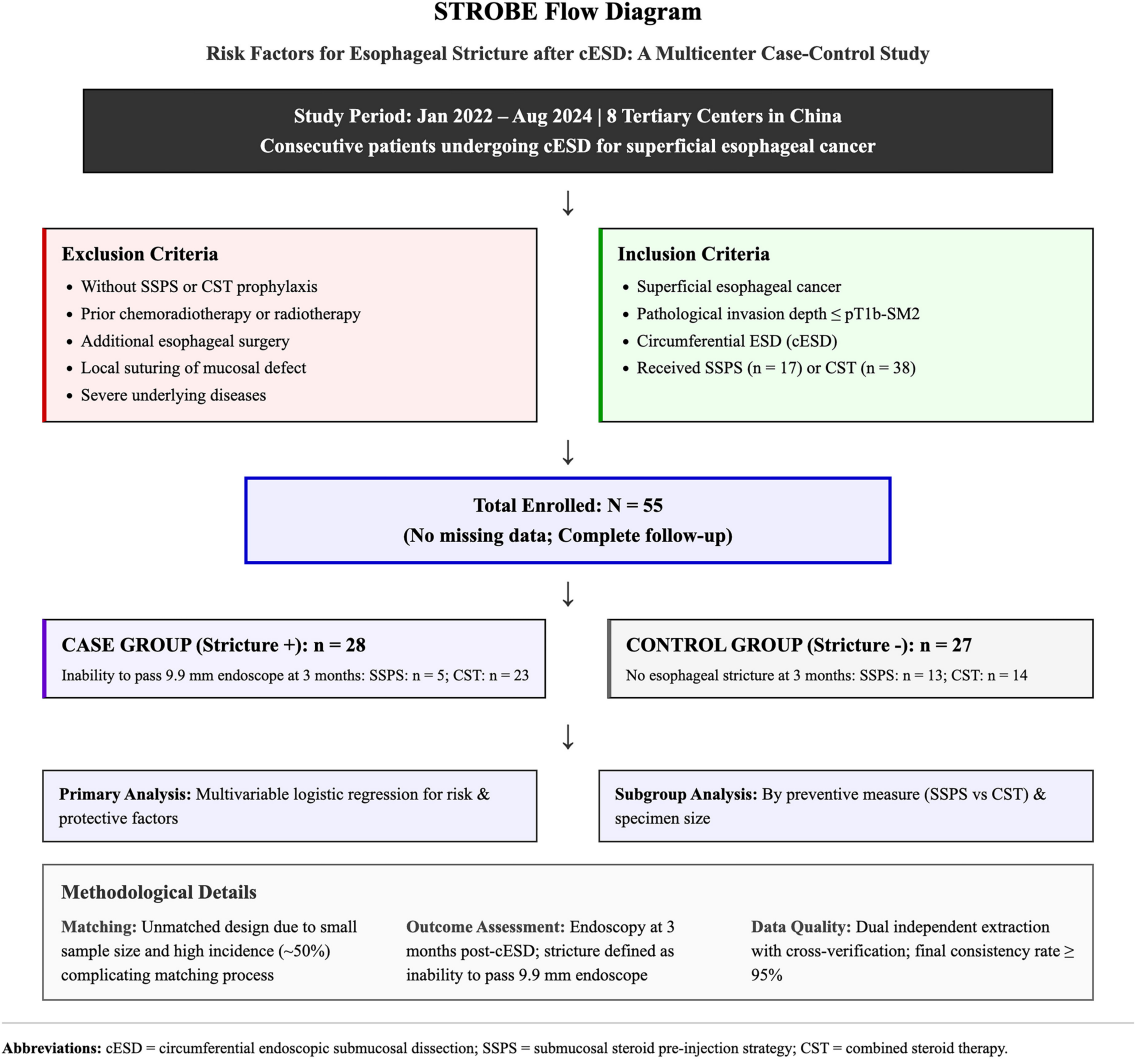
Flow diagram of this case-control study.

Ultimately, 55 patients with superficial esophageal cancer who underwent cESD with either SSPS or CST as the prophylactic measure were enrolled (28 in the stricture group and 27 in the non-stricture group) (Figure 1). Baseline characteristics showed no significant differences in demographic characteristics (sex, age, alcohol consumption, cigarette consumption, family history of esophageal cancer), lesion location, macroscopic types, or pathological invasion depth between the two groups (all *P* > 0.05) (Table 1). The longitudinal length (57.5 [45.0, 78.5] vs. 55.0 [36.5, 68.5] mm; *P* = 0.367) and transverse width (64.4 ± 10.6 vs. 67.4 ± 13.6 mm; *P* = 0.363) of the resected specimen were comparable between the stricture and non-stricture groups. However, the median longitudinal length of circumferential involvement in the stricture group was significantly longer than that in the non-stricture group (60.0 [41.5, 68.5] vs. 40.0 [30.0, 49.0] mm; *P* = 0.045). Additionally, the proportion of patients with a circumferential involvement length over 50 mm was significantly higher in the stricture group than in the non-stricture group (67.9% [19/28] vs. 22.2% [6/27], *P* = 0.002). The utilization rate of SSPS was significantly higher in the non-stricture group than in the stricture group (48.1% [13/27] vs. 17.9% [5/28], *P* = 0.017).

**TABLE 1:** Baseline and cESD characteristics between the stricture and non-stricture groups.

Risk factors	Group	Stricture (*N* = 28)	Non-stricture (*N* = 27)	*P*-value
Demographic characteristics	Sex, female, *n* (%)	8 (28.6)	5 (18.5)	0.576
	Age, mean (SD), range, years	68 (9), (50, 88)	68 (8), (51, 82)	0.847
	Alcohol consumption, *n* (%)	9 (32.1)	12 (44.4)	0.509
	Cigarette consumption, *n* (%)	12 (42.9)	14 (51.9)	0.691
	Family history of esophageal cancer, *n* (%)	3 (10.7)	2 (7.4)	1.000
Lesion features	Lesion location, *n* (%)			
	Lower thoracic	7 (25.0)	8 (29.6)	0.637
	Middle thoracic	14 (50.0)	15 (55.6)	
	Upper thoracic	7 (25.0)	4 (14.8)	
	Macroscopic type, n (%)			
	Depressed	4 (14.3)	4 (14.8)	0.956
	Elevated	6 (21.4)	7 (25.9)	
	Flat	16 (57.1)	13 (48.1)	
	Flat+Depressed	0 (0.0)	1 (3.7)	
	Flat+Elevated	2 (7.1)	2 (7.4)	
	Specimen size, mm#			
	Longitudinal length, median, range, mm	57.5 [45.0, 78.5], (30.0, 110.0)	55.0 [36.5, 68.5], (30.0, 110.0)	0.367
	Longitudinal length > 50 mm, *n* (%)	17 (60.7)	14 (51.9)	0.696
	Transverse width, mean (SD), range, mm	64.4 (10.6), (45.0, 88.0)	67.4 (13.6), (43.0, 95.0)	0.363
	Transverse width > 66 mm, *n* (%)^##^	10 (35.7)	14 (51.9)	0.350
	Longitudinal length of circumferential involvement, median, range, mm^∧^	60.0 [41.5, 68.5], (5.0, 110.0)	40.0 [30.0, 49.0], (10.0, 110.0)	0.045
	Longitudinal length of circumferential involvement > 50 mm, *n* (%)	19 (67.9)	6 (22.2)	0.002
	Pathological invasion depth, n (%)*			
	pT1a-EP	7 (25.0)	4 (14.8)	0.848
	pT1a-LPM	10 (35.7)	10 (37.0)	
	pT1a-MM	5 (17.9)	7 (25.9)	
	pT1b-SM1	5 (17.9)	4 (14.8)	
	pT1b-SM2	1 (3.6)	2 (7.4)	
Prophylactic measures	SSPS, *n* (%)	5 (17.9)	13 (48.1)	0.017
	CST, *n* (%)	23 (82.1)	14 (51.9)	

^#^The longitudinal length and transverse width of the resected specimen were measured at the largest longitudinal and transverse dimensions, respectively. ^∧^The longitudinal length of circumferential involvement was determined by measuring the portion of the longitudinally sliced specimen that encompassed the entire esophageal circumference. *Pathological invasion depth: pT1a-EP: Carcinoma *in situ* (Tis); pT1a-LPM: Tumor invades lamina propria mucosae (LPM); pT1a-MM: Tumor invades muscularis mucosae (MM); pT1b-SM1: Tumor invades the upper third of the submucosal layer; pT1b-SM2: Tumor invades the middle third of the submucosal layer. ^##^The average of transverse width of all included patients was set as the cutoff value.

### Regression analysis

The analysis of risk factors for post-cESD stricture was conducted using both univariate and multivariate statistical methods (Table 2). In the univariate analysis, the longitudinal length of circumferential involvement over 50 mm was significantly associated with an increased risk for post-cESD stricture (OR = 7.39, 95% CI: 2.21–24.66; *P* = 0.001), while SSPS was associated with a reduced risk (OR = 0.23, 95% CI: 0.07–0.80; *P* = 0.020). Other variables did not show a significant association with post-cESD stricture risk in the univariate analysis.

**TABLE 2:** Univariate and multivariate analyses of the risk factors for post-cESD stricture.

Variable	Group	Univariate analysis		Multivariate analysis	
		OR (95% CI)	*P*	OR (95% CI)	*P*
Sex	Female	1.00 (Reference)	0.383		
	Male	0.57 (0.16–2.03)			
Age, years	≤65	1.00 (Reference)	0.612		
	>65	0.74 (0.23–2.38)			
Alcohol consumption	No	1.00 (Reference)	0.349		
	Yes	0.59 (0.20–1.77)			
Cigarette consumption	No	1.00 (Reference)	0.505		
	Yes	0.70 (0.24–2.02)			
Family history of esophageal cancer	No	1.00 (Reference)	0.671		
	Yes	1.50 (0.23–9.76)			
Lesion location	Lower thoracic	1.00 (Reference)	1.00 (Reference)
	Middle thoracic	1.07 (0.31–3.72)	0.919	0.97 (0.21–4.44)	0.965
	Upper thoracic	2.00 (0.41–9.84)	0.394	2.86 (0.34–23.91)	0.331
Longitudinal length, mm	≤50	1.00 (Reference)	0.508		
	>50	1.44 (0.49–4.18)			
Transverse width, mm^##^	≤66	1.00 (Reference)	0.230	1.00 (Reference)	0.359
	>66	0.52 (0.18–1.52)		1.93 (0.47–7.86)	
Longitudinal length of the circumferential involvement, mm	≤50	1.00 (Reference)	0.001	1.00 (Reference)	0.002
	>50	7.39 (2.21–24.66)		9.10 (2.27–33.30)	
Macroscopic type	Depressed	1.00 (Reference)			
	Elevated	0.86 (0.15–5.00)	0.864		
	Flat	1.23 (0.26–5.90)	0.795		
	Flat+Depressed	0.00 (0.00–Inf)	0.991		
	Flat+Elevated	1.00 (0.09–11.03)	1.000		
Pathological invasion depth*	pT1a-EP	1.00 (Reference)			
	pT1a-LPM	0.57 (0.13–2.58)	0.467		
	pT1a-MM	0.41 (0.08–2.19)	0.296		
	pT1b-SM1	0.71 (0.12–4.32)	0.714		
	pT1b-SM2	0.29 (0.02–4.24)	0.363		
Prophylactic measure	CST	1.00 (Reference)	0.020	1.00 (Reference)	0.036
	SSPS	0.23 (0.07–0.80)		0.22 (0.05–0.91)	

^##^The average of transverse width of all included patients was set as the cutoff value. *Pathological invasion depth: pT1a-EP: Carcinoma *in situ* (Tis); pT1a-LPM: Tumor invades lamina propria mucosae (LPM); pT1a-MM: Tumor invades muscularis mucosae (MM); pT1b-SM1: Tumor invades the upper third of the submucosal layer; pT1b-SM2: Tumor invades the middle third of the submucosal layer.

The multi-factor logistic regression analysis confirmed that a longitudinal length of circumferential involvement over 50 mm retained its role as an independent risk factor (OR = 9.10, 95% CI: 2.27–33.30, *P* = 0.002), and SSPS continued to demonstrate a significant protective effect against post-cESD stricture formation (OR = 0.22, 95% CI: 0.05–0.91, *P* = 0.036).

### Subgroup analysis

Subgroup analyses stratified by prophylactic measures (SSPS vs. CST) demonstrated that a longitudinal length of circumferential involvement over 50 mm remained significantly associated with post-cESD stricture in patients receiving CST (*P* = 0.012), whereas this association was absent in the SSPS group (*P* = 0.268) (Table 3). The observed trend suggests that SSPS may weaken the risk effect of extensive circumferential involvement on the formation of post-cESD esophageal stricture.

**TABLE 3:** Subgroup analyses stratified by prophylactic measures.

Group (SSPS or CST)	SSPS			CST		
Group (stricture or non-stricture)	Stricture (*N* = 5)	Non-stricture (*N* = 13)	*P*-value	Stricture (*N* = 23)	Non-stricture (*N* = 14)	*P*-value
Specimen size, mm#						
Longitudinal length, median, mm	66.0 [65.0, 73.0]	55.0 [45.0, 69.0]	0.430	55.0 [43.5, 80.5]	52.5 [32.8, 63.8]	0.397
Longitudinal length > 50 mm, *n* (%)	4 (80.0)	7 (53.8)	0.596	13 (56.5)	7 (50.0)	0.963
Transverse width, mean (SD), mm	60.2 (10.4)	65.9 (16.2)	0.484	60.0 (10.6)	68.8 (11.2)	0.353
Transverse width > 66 mm, *n* (%)^##^	2 (40.0)	6 (46.2)	1.000	8 (34.8)	8 (57.1)	0.322
Longitudinal length of circumferential involvement, median, mm^∧^	60.0 [42.0, 60.0]	45.0 [30.0, 45.0]	0.371	60.0 [42.5, 70.0]	39.0 [30.5, 49.5]	0.124
Longitudinal length of circumferential involvement > 50 mm, *n* (%)	3 (60.0)	3 (23.1)	0.268	16 (69.6)	3 (21.4)	0.012

^#^The longitudinal length and transverse width of the resected specimen were measured at the largest longitudinal and transverse dimensions, respectively. ^∧^The longitudinal length of circumferential involvement was determined by measuring the portion of the longitudinally sliced specimen that encompassed the entire esophageal circumference. ^##^The average of transverse width of all included patients was set as the cutoff value.

## Discussion

This multicenter, retrospective, case-control study provides novel insights into risk stratification and prophylactic strategies for post-cESD esophageal stricture. This study is a secondary analysis of the same multicenter dataset reported previously. While the prior cohort study focused on comparing stricture rates between SSPS and CST, the current case-control design specifically identifies independent risk factors for stricture and quantifies the protective effect of SSPS. This complementary analysis provides deeper mechanistic and prognostic insights that were not addressed in the original report (11). Consistent with previous studies (8, 18–20), our primary findings indicate that a longitudinal length of circumferential involvement over 50 mm is an independent risk factor for post-cESD esophageal stricture, with nearly nine-fold increased odds (adjusted OR 9.10, *P* = 0.002) compared with shorter lesions. Consequently, cESD is not recommended in patients with a longitudinal length of circumferential involvement over 50 mm, even when prophylactic treatment for stricture is implemented (14, 15). Conversely, SSPS appeared as a notable independent protective factor (adjusted OR 0.22, *P* = 0.036), indicating an 78% risk decrease compared to CST. Furthermore, subgroup analysis indicated that extensive circumferential involvement correlated with post-cESD esophageal stricture in patients undergoing CST, where this correlation was eliminated in those receiving SSPS. This suggests that SSPS may be beneficial for high-risk lesions typically considered susceptible to refractory post-ESD stricture formation.

**FIGURE 2:**
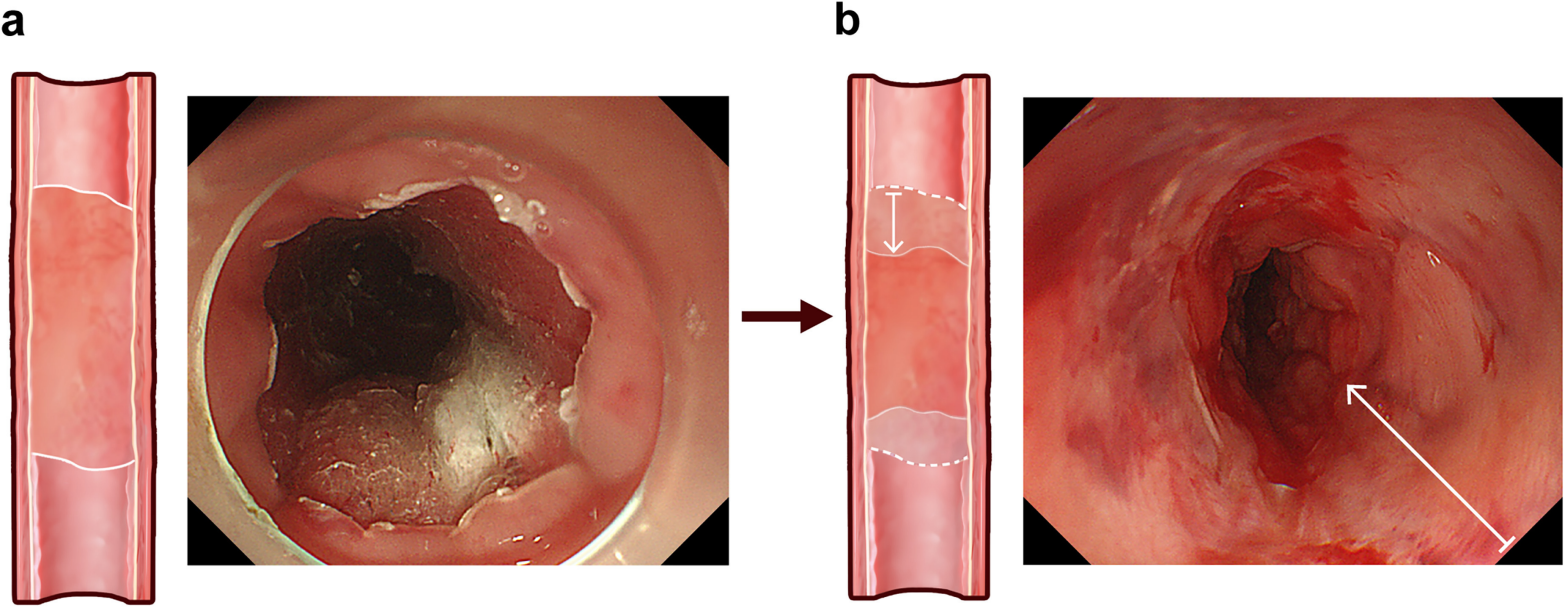
Schematic diagrams and endoscopic images of the epithelialization process in wound healing following cESD. **(a)** The mucosal defect after cESD with white lines demarcating the margins. **(b)** The epithelialization process initiates at the oral and anal margins and progresses inward. The white arrow indicates the direction of epithelialization, and the length represents the extent of the transition band that has already epithelialized.

The clinical issue of post-cESD stricture arises from the anatomical features and distinct wound-healing mechanisms of the esophagus. In contrast to the stomach or colon, the esophagus possesses a relatively narrow lumen and lacks a serosal layer, making it more vulnerable to luminal compromise after extensive ESD (21). Histological investigations have shown that re-epithelialization after cESD proceeds centripetally from the proximal and distal resection margins toward the center of the defect (Figure 2), resulting in esophageal stricture formation in the defect center (22, 23). Our finding that a longitudinal length of circumferential involvement over 50 mm independently predicts stricture risk aligns with this pathophysiological model and corroborates previous observations, identifying the lesion extent as a risk factor for post-cESD esophageal stricture (8, 18–20). These findings emphasize the importance of preoperative lesion evaluation and risk stratification to inform initial treatment and prophylactic strategies.

Steroid-based prophylactic treatments have been widely explored to prevent post-cESD stricture by alleviating inflammation and fibrosis formation in the esophageal tissue. However, steroids administered in the conventional form of oral medication (9, 24, 25) or local injections (5, 26, 27) do not effectively prevent post-cESD stricture, even when given in combination (6–8, 28). We now provide a detailed, reproducible description of SSPS and CST protocols, including steroid dose, concentration, timing, and route of administration. Total steroid exposure was not analyzed due to the retrospective design, but according to the protocols, the steroid dosage in the CST group was significantly higher than that in the SSPS group. SSPS was associated with a superior prophylactic effect with substantially lower total steroid load compared to CST, which is clinically relevant for minimizing systemic steroid-related adverse events.

In both univariate and multivariate analyses, SSPS has been demonstrated as a protective factor against stricture formation. The theoretical foundation for the superior efficacy of SSPS over CST pertains to its multimodal steroid administration. In the creation of the submucosal cushion for lesion elevation and dissection, SSPS involves the pre-injection of TA to ensure immediate and uniform steroid distribution throughout the prospective wound bed, followed by two postoperative intralesional TA injections to maintain therapeutic concentrations during the critical window for fibroblast activation and collagen synthesis (10, 11). Conversely, CST entails postoperative puncture TA injections (leaving gaps), followed by oral prednisone, which restricts steroid distribution and local bioavailability. This pharmacokinetic profile likely elucidated the observed reduction in stricture risk even in high-risk lesions with extensive circumferential involvement over 50 mm. This finding signifies a clinical advance, as lesions of this size have conventionally been recognized as not appropriate for ESD or have mandated aggressive preventive measures, such as prophylactic balloon dilation or biodegradable stent placement, which carry additional costs and procedural risks (29).

Our findings have practical implications for clinical decision-making in patients undergoing cESD. Preoperative assessment of the longitudinal length of circumferential involvement should be routinely performed using high-resolution white-light endoscopy and image-enhanced techniques to identify high-risk lesions (2). For patients with anticipated circumferential involvement over 50 mm, SSPS might be preferentially considered over CST, given its apparent ability to mitigate the substantially elevated stricture risk in this population. Furthermore, our results inform the ongoing debate regarding the optimal management of extensive superficial esophageal cancer. Current guidelines recommend cautious application of cESD for lesions with limited longitudinal extent, even with prophylactic measures (2, 3, 14, 15). The demonstration that SSPS may effectively neutralize the risk associated with more extensive lesions could potentially expand the therapeutic endoscopy armamentarium, offering curative minimally invasive treatment to patients who might otherwise require esophagectomy or definitive chemoradiotherapy with their attendant morbidity.

### Generalizability

The results of this study apply to patients with superficial esophageal cancer (pathological invasion depth ≤ pT1b-SM2) who undergo cESD. For advanced esophageal cancer or patients with severe underlying diseases (such as autoimmune diseases or long-term use of immunosuppressants), the results may not be applicable due to their exclusion from the study, and caution should be exercised when extrapolating. The 8 centers in this study are all tertiary hospitals with mature cESD technology, and the operational consistency is high. Operator experience and center variation may affect the efficacy of SSPS and the incidence of stricture, which were not assessed in this study due to lack of relevant data. For primary hospitals or centers in the early stage of cESD technology development, the implementation effect of SSPS may be affected by differences in operational levels. We recommend conducting standardized training of physicians before promoting SSPS to ensure operational consistency and, consequently, the prophylactic efficacy, especially in Western countries (30).

### Limitation

To our knowledge, this is the largest study regarding the risk factors for post-cESD esophageal stricture; however, it is important to consider several limitations when interpreting the results. First, the relatively small sample size may have diminished statistical power to detect significant associations for certain factors (e.g., pathological invasion depth and macroscopic type) and may increase the risk of overfitting in multivariable analysis. Notably, the subgroup of patients receiving SSPS for lesions with a longitudinal length of circumferential involvement exceeding 50 mm was markedly underrepresented, impeding conclusions about its efficacy in preventing post-cESD esophageal stricture in this high-risk cohort. Second, the retrospective design is inherently susceptible to selection and information biases. Selection bias may have occurred because the choice between SSPS and CST was not randomized but based on physician preference and center-specific protocols. We attempted to control for this issue by adjusting for potential confounders in multivariable analysis, but residual confounding (e.g., operator experience, center variation) cannot be fully excluded. Information bias is possible if endoscopists assessing stricture were aware of the prophylactic strategies used; however, stricture assessment was based on objective criteria (inability to pass a 9.9 mm endoscope), which minimized this risk. Recall bias was limited by using objective medical record data. Third, the non-randomized assignment of treatment constrains causal inference. The unmatched design may have resulted in imbalanced groups, though baseline characteristics were comparable between groups. The non-randomized assignment of treatment constrains causal inference. Forth, the 50 mm cutoff for circumferential involvement length is arbitrary despite being supported by prior studies. Fifth, the subgroup analysis results should be interpreted cautiously given the small sample size of subgroups.

To address these deficiencies, future multicenter prospective studies with large cohorts are imperative, particularly those focused on validating SSPS efficacy in lesions with a longitudinal length of circumferential involvement over 50 mm and clarifying its function in preventing post-cESD esophageal stricture. Furthermore, standardized protocols for outcome evaluation and extended follow-up durations would enhance the clinical relevance of such investigations. Randomized controlled studies comparing SSPS and CST are necessary to confirm the observed prophylactic efficacy.

## Conclusion

This study finds that a longitudinal length of circumferential involvement over 50 mm is an independent risk factor for post-cESD esophageal stricture, while the SSPS serves as a significant protective factor when compared to conventional CST. These findings are preliminary and not yet practice-changing. The SSPS may be integrated into clinical practice for patients needing cESD for superficial esophageal cancer with caution. Notably, SSPS appears to reduce the stricture risk associated with extensive circumferential involvement (exceeding 50 mm), but this observation requires further validation. The potential of SSPS to broaden therapeutic indications for cESD needs to be confirmed in large-scale multicenter prospective trials. Future multicenter prospective trials with large cohorts are essential to validate the prophylactic efficacy and safety of SSPS.

## Data Availability

The original contributions presented in this study are included in this article/supplementary material, further inquiries can be directed to the corresponding authors.
